# Disseminated cryptococcal infection in a lymphoma suspected patient

**DOI:** 10.12669/pjms.38.ICON-2022.5784

**Published:** 2022-01

**Authors:** Nazia Khursheed, Amna Umer, Fareeha Adnan

**Affiliations:** 1 Dr. Nazia Khursheed, FCPS Microbiology, Department of Microbiology, Indus Hospital and Health Network, Karachi, Pakistan; 2 Fareeha Adnan, FCPS Microbiology, Department of Microbiology, Indus Hospital and Health Network, Karachi, Pakistan; 3 Amna Umer, FCPS (Part II Residency), Department of Microbiology, Indus Hospital and Health Network, Karachi, Pakistan

**Keywords:** *Cryptococcus neoformans*, Lymphoma, Sepsis

## Abstract

Cryptococcus neoformans is an opportunistic pathogen, mainly responsible for meningitis in immunodeficient individuals. We report a rare case of dessiminated cryptococcosis in a six years old boy, patient was being evaluated for lymphoma. In the present case the causative agent was *Cryptococcus neoformans*. It was diagnosed through Bactec, aerobic blood culture bottle. The cause of hospitalization of the patient was fever with abdominal pain. Blood and CSF culture revealed the presence of *Cryptococcus neoformans* which was further confirmed by urease test and corn meal tween agar (CMT). In the present case fungus was unusually isolated earlier from blood culture rather than cerebrospinal fluid.

## INTRODUCTION

*Cryptococcus neoformans (C. neoformans)* is a ubiquitous and opportunistic fungal pathogen, primarily acquired via respiratory tract. It is an encapsulated (30 μm thick) unicellular yeast, about 5-12 μm in diameter 1, primarily responsible for cryptococcal meningitis.^1^ According to the Centers for Disease Control and Prevention (CDC), USA, annually there are almost one million new cryptococcal meningitis cases reported worldwide, therefore, is an important global health concern. Clinical case studies suggest that conditions that impair host cell-mediated immunity such as human immunodeficiency virus (HIV) infection^2^ autoimmune diseases, immunosuppressive therapy, liver cirrhosis, lung diseases, lymphoproliferative malignancy and hematological malignancy,^3^ play a central role in the pathogenesis of cryptococcal infections.^4^ These infections with variable clinical presentations have been reported in a variety of immunocompromised patients from different regions of Pakistan^5^ and it is less frequently seen in children than adults.^6^ Herein we report a case of cryptococcosis in a child with lymphoma.

## CASE PRESENTATION

A six years old child from a remote area, presented with a history of intermittent fever, abdominal pain, distension, vomiting, and crusted skin lesions all over his face for 6 months. The symptoms were gradual in onset for which he received certain medications from a local health center and referred to our tertiary care hospital. His previous treatment was unknown. He was semiconscious and tachypneic, therefore admitted to the pediatric intensive care unit. On examination he was icteric, on auscultation, the chest was clear with no murmurs heard, on palpation, abdomen was soft with hepatosplenomegaly and cervical lymphadenopathy.

CT scan with contrast was advised. The imaging appearances of hepatosplenomegaly with cervical, axillary, mediastinal, inguinal and multiple mesenteric lymphadenopathy suggested lymphoproliferative disorder like lymphoma. There was also evidence of left renal and multiple pulmonary deposits. The cervical lymph node was sent for biopsy. It exhibited complete effacement of nodal architecture by sheets of foamy macrophages and multinucleated giant cells along with chronic lymphoplasma cystic infiltrate. Numerous round to oval organisms were seen in the cytoplasm of these foamy macrophages and giant cells, which were highlighted on special stain PAS, GMS, PAS Alcian blue and fontanna masson.

He was treated with teicoplanin 200mg/IV/QDS/24 hrs (10 days), fluconazole, two mg/ml/50ml (14 days), and meropenem 660mg/8 hourly (12 days). CBC reports revealed anisopoikilocytosis, target cells, schistocytes (2%), and large platelets with thrombocytopenia. Procalcitonin was 1.73 ng/ml.

**Fig.1a:**
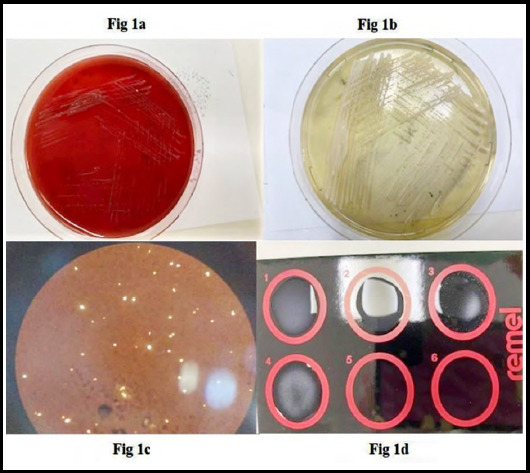
Sheep blood agar, **1b:** SDA, **1c:** India ink, **1d;** (1) negative control, (2) weak positive control, (3) strong positive control, (4) strong agglutination.

Suspecting a fungal infection in immunosuppressed patient Β-d glucan (BDG) was requested which was reported as negative. Simultaneously blood and urine cultures were sent for culture and sensitivity. Urine culture was negative; however aerobic blood culture came positive on the third day of his admission. The gram stain showed rare budding yeast cells. Initially, all the plates were negative only a few small colonies on sheep blood agar grew on the second day of incubation that was not well appreciated (Fig.1a). However, there was no growth on Macconkey and chocolate agar plate. The plates were reincubated for another 24 hrs to confirm the growth of any other pathogen. The colonies observed were creamy and mucoid. The gram stain from colonies showed budding yeast cells. The case was clinically correlated and it was decided to set urease and germ tube test (GTT) on the same day. The urease test was found to be positive within four hours however, slide examination of GTT revealed it to be negative. The same day the sabouraud dextrose agar (SDA) was inoculated. The colony and morphology of yeast on SDA were creamy white with smooth margins (Fig.1b). This was followed by incubation on cornmeal tween agar (CMT). It was interpreted on 3^rd^ day of incubation. Microscopic examination of CMT showed large spherical blastoconidia of different sizes without hyphae. These typical findings confirmed the presence of ***Cryptococcus. Neoformans*:** After reporting *C. neoformans*, the patient was immediately treated with the combination of fluconazole and amphotericin B, 25 mg/IV/24hr for 23 days.

Subsequent blood cultures were done to distinguish between the true fungemia and contamination, but they all turned out to be negative. Meanwhile, the patient developed signs of meningitis. CT brain showed prominent temporal horns of bilateral ventricles hydrocephalous. No abnormality like parenchymal or meningeal enhancement was observed. CT abdomen revealed hepatosplenomegaly with generalized lymphadenopathy and renal deposits. Pulmonary deposits were observed on a chest scan. This was followed by cerebral spinal fluid (CSF) culture. The CSF detailed report showed 29% neutrophils, 65% lymphocytes, 6% monocytes, and budding yeast cells. Numerous RBC were seen because of the traumatic tap. Gram staining, CSF culture, wet prep, and India ink were performed. Donut-shaped yeasts on India ink were seen (Fig.1c). Rapid Cryptococcus antigen test was performed on the CSF sample, with positive and negative controls to assure the presence of yeast, which was also positive (Fig.1d).

The patient was in distress and antifungal fluconazole had already been started. Multiple blood cultures and three consecutive tracheal cultures were sent which turned out to be negative. Histopathology of skin biopsy also revealed the presence of Cryptococcus neoformans.

Initially, he was kept euvolemic. Fundoscopy was done to check papilledema. Patient had an intermittent temperature (37.6 - 39*C) and developed hypernatremia during his stay. He was taken off from the ventilator, once his oxygen level was normalized. No central venous catheter was inserted. Despite aggressive treatment measures the child could not survive and expired within a month of admission.

## DISCUSSION

The threat of cryptococcosis is becoming a global health problem with deadly consequences particularly in immunocompromised individuals.^7^ Cryptococcal infection starts with the inhalation of yeast with the CNS as the main site of infection but it can propagate and cause infection at any site in the body.

In this case *C. neoformans* was isolated from blood culture. It has been observed that it causes fungemia before it crosses the blood brain barrier.^8^ From the present study it is clear that cryptococcus can be isolated from blood in the early phase of dissemination. The 7-day incubation of blood culture bottle at 37*C can facilitate its growth of yeast. The expected reason for earlier detection in blood followed by negative and later on positive CSF culture is that patient might develop sepsis after inhalation of organism which later crossed blood brain barrier and caused meningitis.^8^ In the case of suspected meningitis, CSF and blood cultures should be performed simultaneously. This will enhance the chances of isolation of pathogen at early stage.

## CONCLUSION

This report proposes that in immunocompromised patients cryptococcosis should be considered in the differential diagnosis if there is persistent fever and ineffective antibiotic treatment. Empirical administration of antifungal agent may be necessary. *C. neoformans* antibody test, blood culture, CSF culture and surgical excision biopsy is needed for early diagnosis

### Authors’ Contribution:

**NK** Critically reviewed case report.

**FA** Prepared manuscript draft.

**AU** Information collection and prepared initial draft of manuscript.
